# A comparative study of depression, anxiety, loneliness, well-being and self-esteem among patients with and without Inflammatory Bowel Disease

**DOI:** 10.1192/j.eurpsy.2024.547

**Published:** 2024-08-27

**Authors:** V. Efstathiou, I. Theodoridou, A. Karvouni, E. Kaloudi, P. Bali, A. Papadopoulou

**Affiliations:** ^1^Psychology Department, National and Kapodistrian University of Athens; ^2^Second Department of Psychiatry, National and Kapodistrian University of Athens, “Attikon” University General Hospital, Athens, Greece

## Abstract

**Introduction:**

Individuals diagnosed with Inflammatory bowel disease (IBD) often experience recurring and painful symptoms, which can significantly affect their daily life, while hospitalization and/or surgery may be needed when they present complications. During the course of the disease, IBD patients may experience feelings of anxiety and/or depression and present decreased well-being.

**Objectives:**

The aim of the present study was to investigate depression, anxiety, loneliness, well-being and self-esteem in patients with IBD in comparison to individuals without IBD (healthy controls), while taking into consideration demographic and clinical parameters

**Methods:**

The study included 164 participants and in particular 98 patients with IBD and 66 healthy controls matched for sex and age. All participants completed Hospital Anxiety and Depression Scale (HADS) to assess depression and anxiety, UCLA Loneliness Scale to assess feelings of loneliness, Mental Health Continuum Short Form (MHC-SF) to assess well-being and Rosenberg Self-esteem Scale (RSES) to assess self-esteem, while socio-demographic and clinical data were additionally recorded.

**Results:**

According to the results, statistically significant differences were observed in all psychological parameters with IBD patients presenting higher depression (p<0.001), higher anxiety (p=0.002), higher loneliness (p=0.002), lower well-being (p=0.019) and lower self-esteem (p<0.001) compared to healthy controls. Among IBD patients, higher well-being was independently associated with higher self-esteem and lower anxiety, but not with depression, loneliness or patients’ sex and age.

**Conclusions:**

The findings highlight the importance of investigating and timely detecting psychological symptoms among patients with IBD, with a view to providing them an integrative physical and mental health care.

**Disclosure of Interest:**

None Declared

